# Quantitative Assessment of Pain Threshold Induced by a Single-Pulse Transcranial Magnetic Stimulation

**DOI:** 10.3389/fnins.2020.00559

**Published:** 2020-06-04

**Authors:** Keisuke Tani, Akimasa Hirata, Satoshi Tanaka

**Affiliations:** ^1^Laboratory of Psychology, Hamamatsu University School of Medicine, Shizuoka, Japan; ^2^Department of Electrical and Electronic Engineering, Nagoya Institute of Technology, Aichi, Japan

**Keywords:** pain, transcranial magnetic stimulation, side effect, pain threshold, motor threshold

## Abstract

Transcranial magnetic stimulation (TMS) is commonly used in basic research to evaluate human brain function. Although scalp pain is a side effect, no studies have quantitatively assessed the TMS intensity threshold for inducing pain and whether sensitivity to TMS-induced pain differs between sexes. In the present study, we measured pain thresholds when single-pulse TMS was applied over either Broca’s area (BA) or left primary motor cortex (M1), and compared these thresholds with the motor threshold (MT) for inducing motor evoked potentials (MEPs) through M1 stimulation. Additionally, we compared the pain thresholds for BA and M1 between males and females. We found that pain thresholds for both sites were significantly lower than the MT. Furthermore, the pain threshold for BA was much lower than that for M1. No significant difference was observed between sexes. The results suggest that TMS at an intensity equivalent to MTs, which is often used in experimental or clinical studies, causes slight scalp pain. Experimental designs using TMS to evaluate functional relationships between brain and behavior should consider scalp pain and reduce its likelihood as much as possible.

## Introduction

Transcranial magnetic stimulation (TMS) is widely used in basic research as a tool for evaluating human brain function. Additionally, several studies have demonstrated the effectiveness of repetitive TMS on the recovery of motor, cognitive, or mental function in patients with neurological or psychiatric disorders (e.g., Kobayashi and Pascual-Leone, 2003; Khedr et al., 2010; Ren et al., 2014). In TMS, the strong magnetic field generated by current flowing through the TMS coils induces an electric current in the brain that can lead to a temporary change in brain activity (Rossi et al., 2009). TMS also causes head and face muscles to contract and stimulates cutaneous fibers, which often leads to pain or discomfort on the scalp (Wassermann, 1998; Rumi et al., 2005; Rossi et al., 2009). This side effect of TMS influences aspects of task performance such as accuracy and reaction time (Abler et al., 2005; Meteyard and Holmes, 2018), and to interfere with successful completion of experiments (Wassermann, 1998; Satow et al., 2002). Even though researchers have assessed the degree of pain and the area in which pain is perceived when a certain intensity of TMS is applied (Arana et al., 2008; Meteyard and Holmes, 2018), to our knowledge, no studies have quantitatively evaluated what TMS intensities actually cause pain (i.e., the pain threshold).

In most neurophysiological and neuropsychological studies, TMS intensity is determined based on the motor threshold (MT), which is defined as the minimum current intensity of TMS that produces a pre-defined motor-evoked potential (MEP) amplitude in a target muscle (Rossini et al., 1994). Indeed, an intensity equivalent to 100–120% of MT is often applied in these types of experiments (e.g., Wu et al., 2000; Di Lazzaro et al., 2002). Thus, evaluating whether the pain threshold is higher or lower than MT is critical. The primary purpose of the present study was to measure pain thresholds when single-pulse TMS is delivered to a motor center (primary motor cortex; M1) or a speech motor center (Broca’s area; BA), and compare them with MTs.

While several studies have demonstrated that females have a higher sensitivity to experimental pain stimuli than males (Riley et al., 1998; Andersen et al., 2015), others have showed that pain thresholds do not differ between sexes (Isselee et al., 1998; Racine et al., 2012). In their meta-analysis, Riley et al. (1998) suggested that a large number of participants is necessary to test a sex difference in pain thresholds. In this study, we therefore assessed whether pain thresholds for TMS differed between males and females using a relatively large sample size.

## Materials and Methods

### Participants

The study was approved by the ethical committee of Hamamatsu University School of Medicine and was in accordance with the Declaration of Helsinki. A meta-Analysis (Riley et al., 1998) has shown that 41 participants per group are necessary to assess the sex differences in pain threshold with enough power (*d* = 0.70). Based on this, 82 healthy individuals (41 males and 41 females; 79 Japanese, 1 Vietnamese, and 2 Bangladeshi; age: 26.4 ± 12.3 years) were recruited for the study. All provided written informed consent prior to the experiment. We confirmed through questionnaires that no participants had epilepsy, none had a family history of epilepsy, and none had any neurological or psychiatric disorders. The protocol of this study was pre-registered in the University Hospital Medical Information Network (UMIN) clinical registry (registration number: 000029783) in Japan.

### TMS Device

We delivered single-pulse TMS to the scalp of each participant using a Magstim stimulator (Magstim, 200, Magstim Co. Ltd, United Kingdom) with a figure-eight coil (70-mm diameter; Magstim Co. Ltd, United Kingdom).

### Stimulation Site and Coil Orientation

We applied TMS over the targeted brain locations using neuro-navigation. Before the TMS experiment, each participant underwent a T1-weighted magnetic resonance imaging (MRI) head scan with a 3T scanner (Discovery MR750 3.0T, GE Healthcare Japan, Japan). The scanner setting was as follows: repetition time (TR) = 7.2 ms, echo time (TE) = 2.1 ms, flip angle (FA) = 15°, field of view (FOV) = 25.6 cm^2^, voxel size = 1 mm × 1 mm × 1 mm, and matrix = 256 × 256. Based on the MRI images, we created a 3D cortical surface model of each participant using a frameless navigation system (Brainsight, Rogue Research Inc., Canada). Using this device, we could continuously monitor the position and orientation of a TMS coil relative to a participant’s head by capturing the reflection markers mounted on the head and the coil with a camera. This allowed us to accurately stimulate the regions of interest (left M1 or BA) during the experiment.

In this study, we followed the same procedure as Sparing et al. (2008) in which the stimulation site was anatomically defined as the hand knob, a landmark of the hand motor cortex (Yousry et al., 1997), using a neuro-navigation system. The advantage of this method is that experimenters can determine the target stimulation site more easily and more quickly, which can reduce participant fatigue. For BA, we used Brodmann area 44. The orientation of the magnetic coil for M1 was set as a posterior and lateral handle orientation, 45 degrees relative to the antero-posterior axis of the head, which appears to be the optimal angle for inducing MEPs (Mills et al., 1992). The coil orientation for BA was set as a posterior handle orientation, i.e., parallel to antero-posterior axis of the head, seen from the lateral side.

### Threshold Measurements

We measured the pain thresholds for M1 and BA and the MT for M1 once for each participant. We additionally assessed the perceptual threshold at each stimulation site to confirm whether pain sensation differed from the perception of force or pressure induced by TMS. During all measurements, participants sat on a reclining chair and were asked to relax. The order of stimulation sites was randomized across participants. At each stimulation site, the perceptual thresholds were assessed before the pain thresholds, but the order of perceptual/pain-threshold assessment and MT assessment for M1 was randomized across participants.

We inserted a 5-s pause between each trial. To avoid participant fatigue, we included an approximately 10-min break before switching stimulation sites. If the participants seemed unable to concentrate on the task during the experiment because of fatigue or sleepiness, we inserted additional breaks accordingly. Stimulation at each site lasted about 20–30 min (1 h in total).

#### Perceptual Threshold

After each stimulation, participants were asked to verbally report whether or not they perceived force or pressure on their scalps. Perceptual thresholds were measured using an adaptive staircase method; the intensity was decreased when the participants reported feeling the force, and increased when they reported not feeling any force. Perceptual thresholds were defined as the minimum intensity that induced the sensation of pressure or force in at least 2 of 3 trials; the minimum intensity is represented in terms of percentage of maximum stimulator output (MSO).

#### Pain Threshold

Participants were asked to verbally report the presence or absence of scalp pain after each stimulation. As with the perceptual threshold, we applied an adaptive staircase method to evaluate pain thresholds, which were defined as the minimum intensity that induced pain on the scalp in at least 5 of 10 trials. We proceeded to the next stimulation intensity when we observed positive responses (pain was felt) in at least 5 of 10 trials or negative responses (no pain) in 6 of 10 trials.

#### Motor Threshold

We measured the pain thresholds for of MEPs induced by TMS over left M1 (the hand knob). Electromyography (EMG) of the right first dorsal interosseous muscle (FDI) was recorded using Ag/AgCL surface electrodes (10 mm in diameter). The EMG signals were amplified, bandpass filtered between 16–470 Hz, and sampled at 3 kHz by a Rogue EMG device. During this measurement, participants were asked to keep their hands as relaxed as possible. A researcher carefully confirmed whether the muscles were relaxed by watching the EMG activity on a monitor while applying each stimulation. MTs were measured using an adaptive staircase method and defined as the minimum intensity that induced MEPs whose amplitudes were larger than 50 μV in at least 5 of 10 trials. As with pain thresholds, we proceeded to the next stimulation intensity if we observed positive responses (MEP ≥ 50 μV) in 5 of 10 trials or negative responses (MEP < 50 μV) in 6 of 10 trials.

### Data Analysis

Perceptual and pain thresholds, and MTs were determined for each participant. Shapiro–Wilk tests revealed that threshold values were not normally distributed across participants (*p* < 0.001 for BA, *p* < 0.01 for M1). Therefore, we used non-parametric tests to compare these thresholds. We first compared perceptual and pain thresholds for each stimulation site using Mann–Whitney *U* tests. Subsequently, we compared the pain thresholds for BA and M1 and the MTs using a Friedman test followed by post-hoc Scheffé tests. We also compared pain thresholds for BA and M1 between males and females using Mann–Whitney *U* tests.

## Results

**FIGURE 1:**
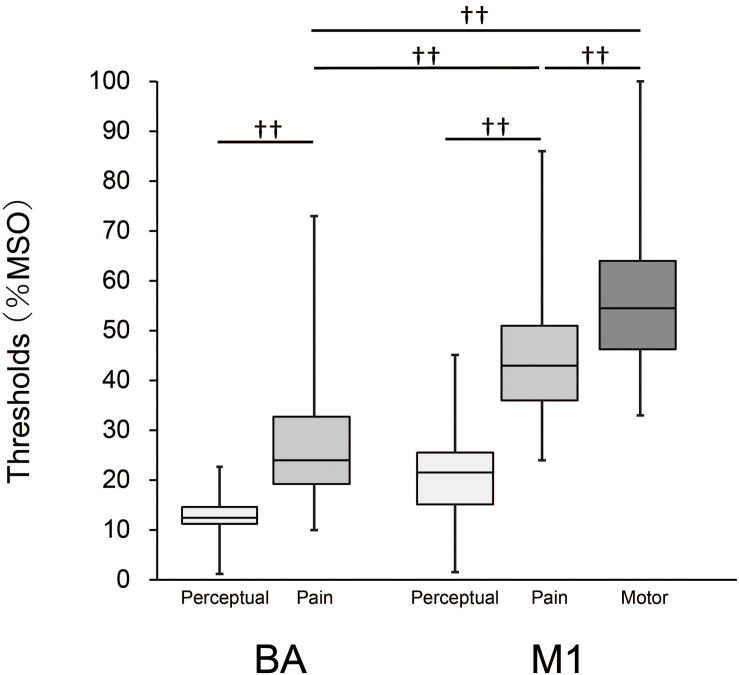
Box-whisker plots for perceptual and pain thresholds and MT. Each box covers the range between the 1st and 3rd quartiles of each threshold. The horizontal line within the box represents the median value for each threshold. Upper and lower ends of each whisker represent the maximum and minimum values for each threshold, respectively. ^††^, *p* < 0.001.

The result for each threshold is presented in Figure 1. Median (1st, 3rd quartiles) for perceptual thresholds were 12.5% (15.0, 11.0) for BA and 21% (25.0, 16.0) for M1, and those for pain were 24.0% (32.8, 19.3) for BA and 43.0% (51.0, 36.0) for M1. The median MT was 54.5% (64.0, 46.3). The minimum value of pain thresholds for BA and M1 were 10% and 24%, respectively. Mann–Whitney tests showed that the perceptual thresholds were significantly lower than the pain thresholds for both stimulation sites (BA: *z* = 9.3, *p* < 0.001; M1: *z* = 10.4, *p* < 0.001), indicating that the sensation of pain was clearly different from that of force or pressure. A Friedman test revealed a significant main effect of threshold between the pain thresholds for BA and M1 and the MT [χ^2^_(2)_ = 112.2, *p* < 0.001]. Moreover, post-hoc tests showed that the pain threshold for BA was significantly lower than both the pain threshold for M1 and the MT (both *p* < 0.001), and that the pain threshold for M1 was significantly lower than the MT (*p* < 0.001). The pain thresholds for BA and M1 were lower than the MT in 78 (95%) and 67 participants (82%), respectively.

**FIGURE 2:**
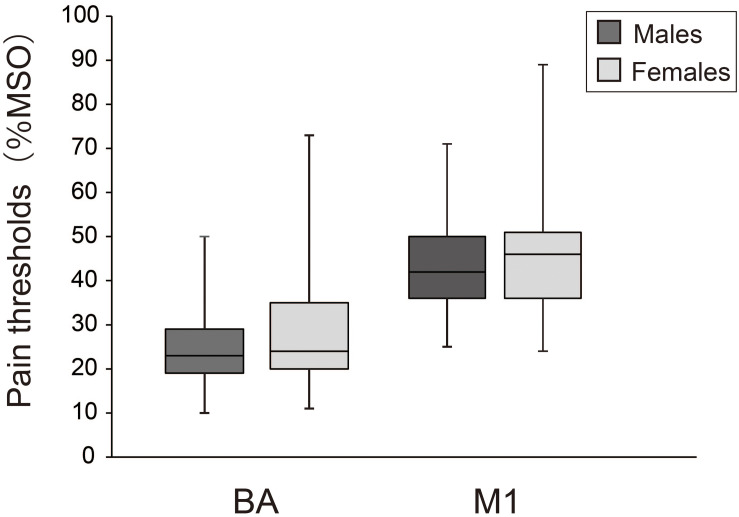
Box-whisker plots of pain thresholds for BA and M1 in males and females.

Figure 2 compares pain thresholds for males and females. Median (1st, 3rd quartiles) pain thresholds for males and females were 23.0% (29.0, 19.0) and 24.0% (35.0, 20.0) for BA, and 42.0% (50.0, 36.0) and 46.0% (51.0, 36.0) for M1, respectively. Mann–Whitney *U* tests revealed no significant differences between sexes for either BA (*z* = 0.79, *p* = 0.43) or M1 (*z* = 0.60, *p* = 0.55).

## Discussion

In most clinical or experimental studies using TMS, the intensity of stimulation is determined as equivalent to or above individual MTs, but whether stimulation at this intensity causes scalp pain is unclear. The present study quantitatively evaluated pain thresholds for single-pulse TMS delivered to M1 and BA, and compared them with MT. We found that on average, pain thresholds for both BA and M1 were much lower than the MT. Individually, pain thresholds for both sites were lower than the MT in more than 80% of participants. These results indicate that participants feel slight pain when TMS is applied at intensities equivalent to the MT.

It has been demonstrated that pain or discomfort induced by TMS influences task performance (Abler et al., 2005; Holmes and Meteyard, 2018; Meteyard and Holmes, 2018). For instance, Abler et al. (2005) showed that the error rate on a visual memory task increased with the magnitude of subjective discomfort induced by TMS. Based in these findings, we must consider that any temporary changes in motor or cognitive performance observed after applying TMS to a particular brain region might also be related to the side effect of pain. Therefore, to properly evaluate the functional relationships between brain and behavior using TMS, we need to design experiments in which the influence of pain sensation is controlled for. This could take the form of a sham condition in which the same degrees of pain sensation are caused on the scalp.

We also found that pain thresholds were much lower for BA than for M1. This difference in pain threshold between sites could be related to anatomical differences in muscle volume. While the tissues of temporalis muscle are thickly distributed on the scalp above BA, aside from the epicranial aponeurosis, muscle tissues are not distributed above M1 (Netter, 2014). Based on this fact, the present result proposes that TMS-induced pain can be attributed primarily to muscle twitches rather than activation of cutaneous nerves. Indeed, some studies have suggested that magnetic stimulation can stimulate the peripheral or central neuromuscular system without strongly activating skin nociceptors (Barker et al., 1987; Barker, 1991; Han et al., 2006). TMS over BA would strongly stimulate the muscle nociceptors (i.e., free nerve endings of Aδ or C fibers), which would result in easy elicitation of pain.

In the present study, the MTs (54.5%) were relatively higher than what has been reported previously (Rossini et al., 2015; 35%–44%). One reason for this might be the methodological difference in how the target location in M1 was determined. While the previous studies used TMS and the hotspot method to determine the target M1, here we used a neuro-navigation system to define it anatomically as the hand knob (Sparing et al., 2008). However, an anatomically-defined hand knob appears to be spatially inconsistent with MEP hotspots found using TMS. Studies have shown that the hand knob and the hotspot were near each other, but not completely in line (Ahdab et al., 2010, 2016). Thus, this difference in location likely results in an overestimation of MTs when the hand knob is used and might explain the high MTs that we observed in the present study. Indeed, when Sparing et al. (2008) investigated whether MEP size differed depending on how the target M1 was defined, they found that MEPs induced by TMS over the hand knob were 20% smaller than those induced by TMS over the hotspot. Based on this finding, MTs at the hotspot examined by conventional TMS methods can be estimated as 43.6% in the present study, which is the same as or a little higher than the pain threshold for M1 (43.0%) and much higher than the pain threshold for BA (24.0%). Thus, even if the MTs were measured based on the hotspot using conventional TMS methods, MTs for M1 and BA would still be expected to be the same or higher than pain thresholds.

An alternative or additional reason for the higher MTs in this study could be related to the racial makeup of participants among the studies. A TMS study (Yi et al., 2014) showed that the resting MTs in Han Chinese (50%) was significantly higher than the MTs in Caucasian European (40%). This finding suggests that the MTs in Asian people (including Japanese the majority of participants in the present study) might be higher than those in Caucasian European, possibly because of the differences in skull shape between these races (Yi et al., 2014).

One limitation of the present study is that we only measured the MTs for one muscle (FDI). The amplitude of MTs is known to depend on the type of target muscle. MTs typically tend to be higher for proximal muscles than for distal hand muscles (Rossini et al., 2015). Therefore, if more proximal muscles (e.g., the flexor carpi radialis muscle) were targeted, clearer differences could be observed between the pain thresholds for M1 and MTs.

In addition, we only subjectively measured pain thresholds. The details of how TMS stimulates each tissue within the scalp (skin, fat, or muscle) remains unclear. Thus, the present result cannot exclude the possibility that the activation of peripheral nerves also contributes to pain sensation. Skin thickness seems to vary depending on cranial sites (Young, 1959), which might partially explain the differences in pain thresholds between the cortical sites (Rashed et al., 2019). To clarify the mechanism of pain sensation, further computational studies are required to estimate the distribution of the electric field over skin and muscle tissues induced by TMS.

In conclusion, the present study shows that even though its sensation is relatively weak around pain thresholds, TMS induces pain at intensities equivalent to MTs, especially over the BA. Therefore, researchers who study TMS need to remind themselves that some participants might feel pain even when TMS intensity is lower than the MTs in their experiments.

## Data Availability Statement

The datasets generated for this study are available on request to the corresponding author.

## Ethics Statement

The studies involving human participants were reviewed and approved by the ethical committee of Hamamatsu University School of Medicine. The participants provided their written informed consent to participate in this study.

## Author Contributions

ST and AH developed the study concepts and designed the experiments. KT performed the experiments and analyzed the data. KT and ST wrote the manuscript.

## Conflict of Interest

The authors declare that the research was conducted in the absence of any commercial or financial relationships that could be construed as a potential conflict of interest.
